# In-hospital fall prediction using machine learning algorithms and the Morse fall scale in patients with acute stroke: a nested case-control study

**DOI:** 10.1186/s12911-023-02330-0

**Published:** 2023-11-01

**Authors:** Jun Hwa Choi, Eun Suk Choi, Dougho Park

**Affiliations:** 1 College of Nursing, Kyungpook National University, 680 Gukchaebosang-ro, Jung-gu, Daegu, 41944 Republic of Korea; 2 Department of Quality Improvement, Pohang Stroke and Spine Hospital, Pohang, Republic of Korea; 3 Research Institute of Nursing Science, Kyungpook National University, Daegu, Republic of Korea; 4 Medical Research Institute, Pohang Stroke and Spine Hospital, 352, Huimang-daero, Nam-gu, Pohang, 37659 Republic of Korea; 5 Department of Medical Science and Engineering, School of Convergence Science and Technology, Pohang University of Science and Technology, Pohang, Republic of Korea

**Keywords:** Accidental falls, Machine learning, Risk assessment, Stroke

## Abstract

**Background:**

Falls are one of the most common accidents in medical institutions, which can threaten the safety of inpatients and negatively affect their prognosis. Herein, we developed a machine learning (ML) model for fall prediction in patients with acute stroke and compared its accuracy with that of the existing fall risk prediction tool, the Morse Fall Scale (MFS).

**Methods:**

This is a retrospective nested case-control study. The initial sample size was 8462 admitted to a single cerebrovascular specialty hospital with acute stroke. A total of 156 fall events occurred, and each fall case was randomly matched with six control cases. Six ML algorithms were used, namely, regularized logistic regression, support vector machine, naïve Bayes (NB), k-nearest neighbors, random forest, and extreme-gradient boosting (XGB).

**Results:**

We included 156 in the fall group and 934 in the non-fall group. The mean ages of the fall and non-fall groups were 68.3 (± 12.2) and 65.3 (± 12.9) years old, respectively. The MFS total score was significantly higher in the fall group (54.3 ± 18.3) than in the non-fall group (37.7 ± 14.7). The area under the receiver operating curve (AUROC) of the MFS in predicting falls was 0.76 (0.73–0.79). XGB had the highest AUROC of 0.85 (0.78–0.92), and XGB and NB had the highest F1 score of 0.44.

**Conclusions:**

The AUROC values of all of ML algorithms were similar to those of the MFS in predicting fall risk in patients with acute stroke, allowing for accurate and efficient fall screening.

**Supplementary Information:**

The online version contains supplementary material available at 10.1186/s12911-023-02330-0.

## Background

In-hospital falls are among the most common patient safety incidents in healthcare facilities [1]. They can increase the length of hospital stay, incur additional healthcare costs, and can even lead to legal disputes between the healthcare providers and patients [2]. In their multi-center study, Morello et al. [3] found that in-hospital falls cause an average of eight additional days of stay in hospital and incur an average additional cost of $6669. Furthermore, they negatively affect patients and their families because of increased time and financial burden [4]. The incidence of falls is particularly high among those with cerebrovascular diseases due to impaired postural stability, decreased sensory function, and motor deficits [5]. Previous studies have reported high post-stroke fall rates, with 1.8–14% of patients with stroke experiencing falls during hospitalization [6, 7].

Medical staff must assess fall risk based on a patient’s characteristics to effectively predict their probability [8]. There have been several fall risk assessing tools, such as the St. Thomas Risk Assessment Tool [9], Hendrich II Fall Risk Model [10], Johns Hopkins Fall Risk Assessment Revised Tool [11], and Morse Fall Scale (MFS) [12]. These tools have been developed by determining and categorizing the fall risk factors. However, sufficient staff and time are required to complete these evaluations and they do not sufficiently reflect the characteristics of those patients with potential risks [13, 14]. Among them, the MFS is the most widely utilized tool for assessing the risk of falls in South Korea [13]; it consists of six items, including fall history, secondary diagnosis, use of assistive devices, intravenous or heparin cap, gait, and self-insight related to gait disorders [12, 15]. The MFS has been validated in several studies and is considered a reliable tool for measuring fall risk [16–18]. However, it has limitations in predicting fall risk factors in uncooperative patients. Therefore, reflecting the characteristics of a patient’s clinical situation in fall prediction and supplementing the drawbacks or limitations of fall risk assessment tools used in clinical practice are needed to improve fall prediction [19, 20]. Various factors affect the likelihood of falls and, in medical institutions, which treat patients with severe disease, a predictive model for disease-specific fall risk factors is essential. Nevertheless, fall risk screening tools are not sufficient to prevent in-hospital falls [14].

In recent years, there has been a rapid increase in medical research based on machine learning (ML) [21]. It is primarily used for implementing prediction models; however, its scope is expanding to include the classification of disease severity [22], medical decision-making [23], and application of newly developed therapeutic interventions [24]. An advantage of ML-based models is their ability to predict a patient’s prognosis or progress in a specific situation based on data from the electronic health records (EHRs) [25]. ML is able to integrate clinical information in a meaningful manner, providing medical staff with comprehensive information for ensuring fully informed medical decision-making [26]. Previous studies have shown that ML-based algorithms can produce results equivalent to, or better than, those produced by traditional tools if sufficient data and appropriate algorithms are used [27, 28]. However, to the best of our knowledge, there have been no studies till date presenting an ML-based fall prediction model in hospitalized patients with acute stroke.

This study aimed for the following: (1) to develop an ML model for prediction of in-hospital fall risk among patients with acute stroke; (2) to compare the ML model’s predictive performance with that of the existing fall risk assessment tool, the MFS.

## Methods

### Data source and patient inclusion

**Fig. 1:**
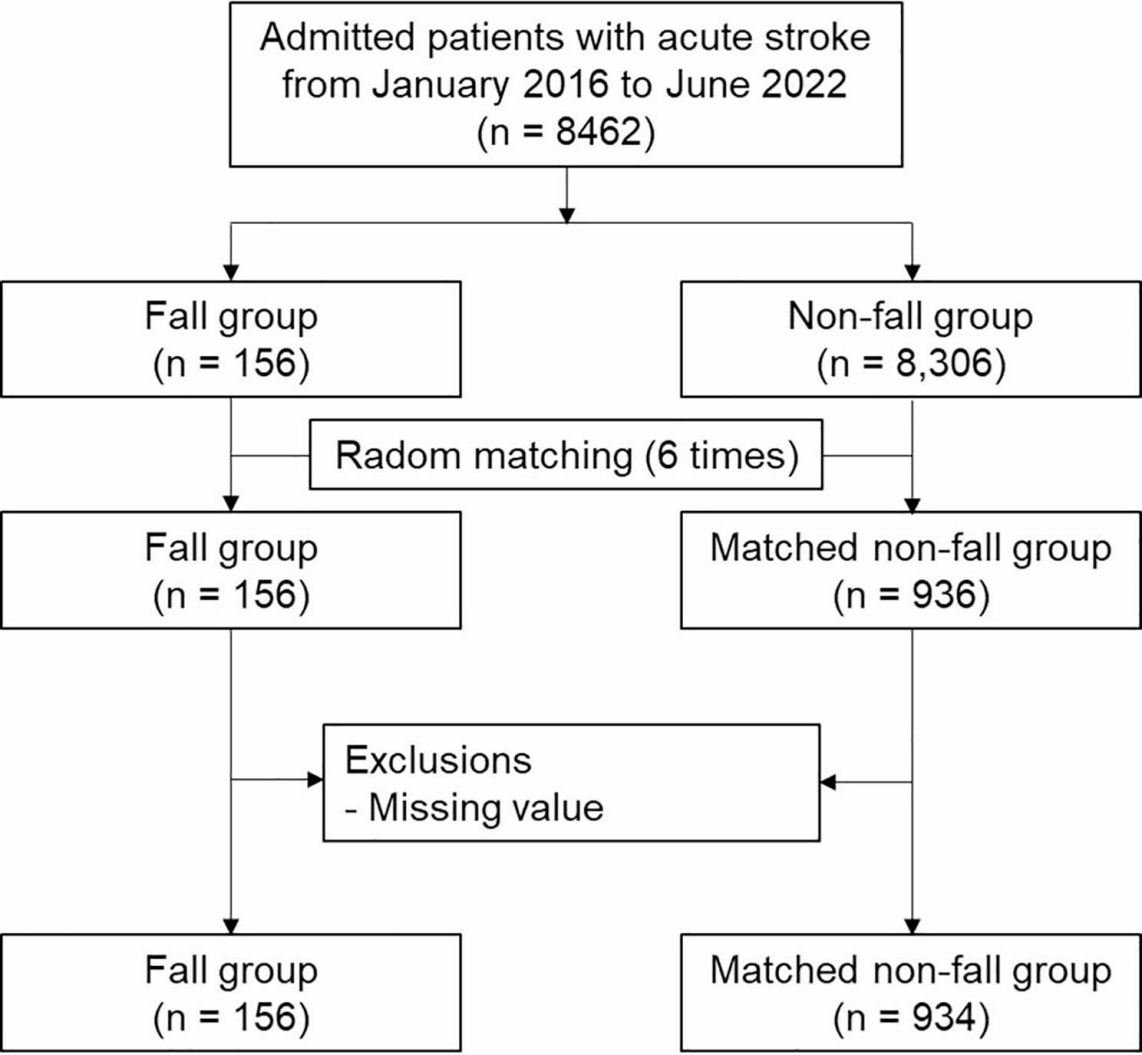
Flowchart of patient inclusion

This retrospective study utilized EHRs to identify patients who were admitted to a single cerebrovascular specialty hospital between January 2016 and June 2022 with a primary diagnosis of acute stroke, as defined by the International Classification of Diseases-10 codes I60–I63. We initially identified 8462 patients. During this period, 156 fall events occurred (1.84%). If a significant difference in frequency was found between the fall event group and the control group, a retrospective nested case-control study was performed using random sampling methods, which were frequently used in previous related studies [20, 29]. Each fall case was randomly matched with six control groups (n = 936), with matching performed based on admission in the same quarter and ward. Cases with missing values were excluded from the study (Fig. 1). For the robustness of the statistical analysis, if there were two or more fall events in one admission, the first fall was used as the index.

This study design was reviewed and approved by the Institutional Review Board of Pohang Stroke and Spine Hospital (Approval No. PSSH0475-202108-HR-016-04). The informed consent was waived by the Institutional Review Board of Pohang Stroke and Spine Hospital due to the retrospective nature of this study and anonymity of the database. The study was conducted following the principles of the Declaration of Helsinki.

### Study variables

Evaluation indicators assessed during the initial hospitalization were used as the main variables to predict falls. Age and sex were identified as basic information. Body mass index (BMI), haemoglobin level, and albumin level were checked to reflect the patient’s nutritional status. Stroke subtypes were classified as subarachnoid hemorrhage (I60), intracerebral hemorrhage (I61 and I62), or ischemic stroke (I63). Finally, the National Institutes of Health Stroke Scale (NIHSS) score was assessed to identify stroke severity.

As factors reflecting the patient’s status at admission, the differences in admission route, admission method, and ward type were assessed. As a result, the admission route was divided into emergency room and outpatient admission, and the admission method was classified into walking, wheelchair, and bedridden. In addition, the initial admission ward was classified into general ward, integrated nursing care service (INCS), and special care units – intensive care unit (ICU) and stroke unit (SU).

Socioeconomic factors were divided into the medical insurance type and residence area. The medical insurance type was classified into medical aid and national health insurance coverage. According to the Korean administrative distinct, the residential area was divided into the “dong” and “*eup/myeon*.” Accompanying diseases such as hypertension, diabetes, dyslipidemia, arrhythmia, cardiovascular diseases, osteoporosis, degenerative spinal disease, and neurodegenerative brain disease were assessed (Table S1). The prescribed drugs were checked with the standard drug code name and the Anatomical Therapeutic Chemical Classification System developed by the World Health Organization. In the fall group, drugs administered on the day of the fall event were included, and in the control group, drugs administered at the time of admission were included. Medications were categorized into antidepressants, anxiolytics, antipsychotics, antiepileptic drugs, and diuretics, and patients were classified as those without medication history in the category, those taking only one type of medication, and those taking multiple classes of medications.

Finally, our ML models were compared with the existing fall risk prediction tool, the MFS, which was evaluated by skilled nursing staff at the time of patient admission. The total MFS scores, a routine assessment of fall screening in the setting of this study, was used to predict falls. The list of all variables used in the predictive model is summarized in Table S2.

### Statistical analysis

This study used the R software version 4.3.0 (R Core Team, R Foundation for Statistical Computing, Vienna, Austria) for all statistical and ML analyses. Continuous variables were presented as mean ± standard deviation, and categorical variables were presented as frequencies (percentages). For comparison between the fall and non-fall groups, independent t-tests were performed for continuous variables, and chi-square (trend) tests were performed for categorical variables. *P*-values of < 0.05 were considered statistically significant. Univariable binary logistic regression models were applied to evaluate the predictive power of the MFS, and the area under the receiver operating characteristic curve (AUROC) was calculated and compared with other ML models.

To investigate the relationship between fall occurrence and variables, a binary logistic regression model was established. Variable selection was performed using stepwise backward elimination, and the Akaike information criterion was used as an estimator of multivariable model fitness. The measurement of multicollinearity was conducted using the criterion of sqrt (variable inflation factor) > 2.

### Data pre-processing and ML process

**Fig. 2:**
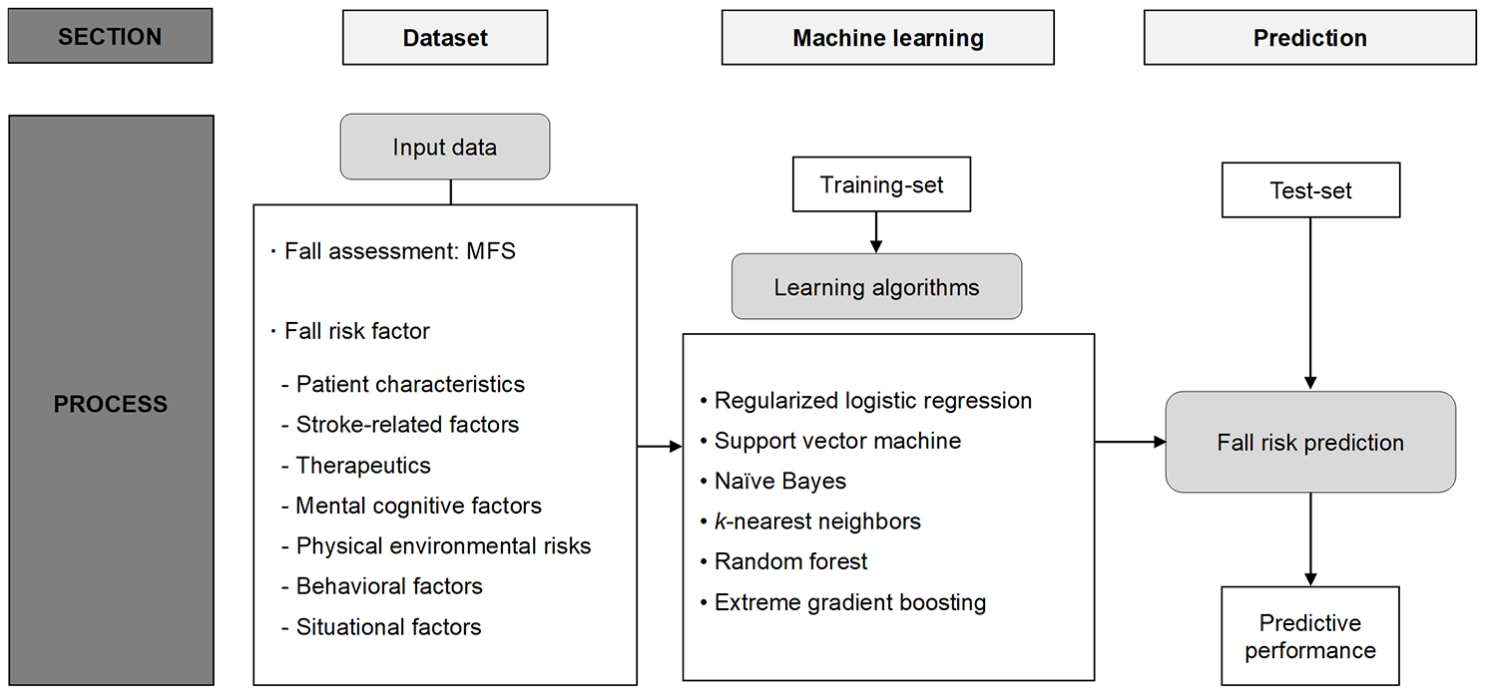
Frame of machine learning prediction for this study

Data pre-processing was performed for the ML prediction model. First, variables with low frequency and those showing multicollinearity were identified. For continuous variables, centering and scaling were performed. One-hot encoding was applied to convert categorical variables into numeric variables. Data were randomly divided into training and validation data at a 2:1 ratio. To balance the dependent variable, training data were oversampled using the synthetic minority oversampling technique. Six ML algorithms were used for the ML process, namely, regularized logistic regression (RLR), support vector machine (SVM), naïve Bayes (NB), k-nearest neighbors (KNN), random forest (RF), and extreme-gradient boosting (XGB). For internal validation, 10-fold cross-validation was repeated 50 times using training data. Hyperparameter tuning was conducted using a combination of random and grid searches (Table S3). To assess their prediction performance in terms of AUROC, F1 score, sensitivity, specificity, positive predictive value, and negative predictive value, the optimal trained models for each algorithm were applied to the validation data. Finally, feature importance was measured for the RLR, RF, and XGB models (Fig. 2). The “caret” package in R software was used for the ML process [30]. The entire code for this study is provided in the online supplementary materials.

## Results

### Baseline characteristics and the Morse fall scale

In our final analysis, there were 156 and 934 patients in the fall and non-fall groups, respectively. Table 1 summarizes the baseline characteristics of the patients in the fall and non-fall groups. The features of the in-hospital falls recorded are summarized in Table 2.

**Table 1:** Baseline characteristics

Variables	Non-Fall (n = 934)	Fall (n = 156)	*p*-value
Age, years old	65.3 ± 12.9	68.3 ± 12.2	0.063
Body mass index, kg/m^2^	23.8 ± 3.1	23.2 ± 3.1	0.041
Male, n (%)	566 (60.0)	80 (51.3)	0.035
Hemoglobin, g/dL	12.8 ± 2.0	12.5 ± 1.8	0.045
Albumin, g/dL	3.9 ± 0.5	3.8 ± 0.5	0.373
Ward type, n (%)			< 0.001
General	433 (46.4)	87 (55.8)	
Integrated nursing care service	467 (50.0)	41 (26.3)	
Intensive care unit/stroke unit	34 (3.6)	28 (17.9)	
Insurance type, n (%)			0.282
National health insurance covered	865 (92.6)	140 (89.7)	
Medical aid	69 (7.4)	16 (10.3)	
Residential area, n (%)			0.377
Urban	416 (44.5)	76 (48.7)	
Rural	518 (55.5)	80 (51.3)	
Stroke subtype, n (%)			< 0.001
Subarachnoid hemorrhage	99 (10.6)	16 (10.2)	
Intracranial hemorrhage	120 (12.8)	36 (23.1)	
Ischemic	715 (71.6)	104 (66.7)	
Admission route, n (%)			0.002
Emergency department	581 (62.2)	118 (75.6)	
Outpatient clinic	353 (37.8)	38 (24.4)	
Admission state, n (%)			< 0.001
On foot	363 (38.9)	34 (21.8)	
Wheelchair	218 (28.3)	44 (28.2)	
Bed	353 (37.8)	78 (50.0)	
Hypertension, n (%)	515 (55.1)	99 (63.5)	0.064
Diabetes, n (%)	229 (24.5)	47 (30.1)	0.164
Dyslipidemia, n (%)	369 (39.5)	48 (30.8)	0.047
Arrhythmia, n (%)	59 (6.3)	24 (15.4)	< 0.001
Coronary artery disease, n (%)	41 (4.4)	6 (3.8)	0.923
Osteoporosis, n (%)	27 (2.9)	8 (5.1)	0.222
Degenerative spinal diseases, n (%)	26 (2.8)	15 (9.6)	< 0.001
Cerebral neurodegenerative diseases, n (%)	38 (4.1)	20 (12.8)	< 0.001
Medication, n (%)			< 0.001
None	511 (54.7)	52 (33.3)	
Single	321 (34.4)	82 (52.6)	
Poly	102 (10.9)	22 (14.1)	
Alert mental status, n (%)	792 (84.8)	134 (85.9)	0.814
Morse Fall Scale total	37.7 ± 14.7	54.3 ± 18.3	< 0.001
NIHSS	4.0 ± 6.2	5.3 ± 4.1	< 0.001

NIHSS, National Institutes of Health Stroke Scale

**Table 2:** Features of in-hospital falls in this study

Variables	n (%)
Place	
Room	114 (73.1)
On the move	8 (5.1)
ER/ICU/SU	21 (13.5)
Others	13 (8.3)
Injury	
None	117 (75.0)
Abrasion/laceration	27 (17.3)
Hematoma	4 (2.6)
Fracture	7 (4.5)
Head trauma	1 (0.6)
Time	
Working time (8:00–19:00)	61 (39.1)
Day	
Weekday (Monday–Friday)	108 (69.2)

ER, emergency department; ICU, intensive care unit; SU, stroke unit

The mean MFS score was significantly higher in the fall group (54.3 ± 18.3) than in the non-fall group (37.7 ± 14.7). The AUROC of the MFS in predicting falls was 0.76 (0.73–0.79), and the sensitivity and specificity were 0.72 (0.65–0.79) and 0.74 (0.71–0.77), respectively. The cutoff value for predicting falls using the mean MFS score was 42.50 points.

### Stepwise logistic regression model

**Fig. 3:**
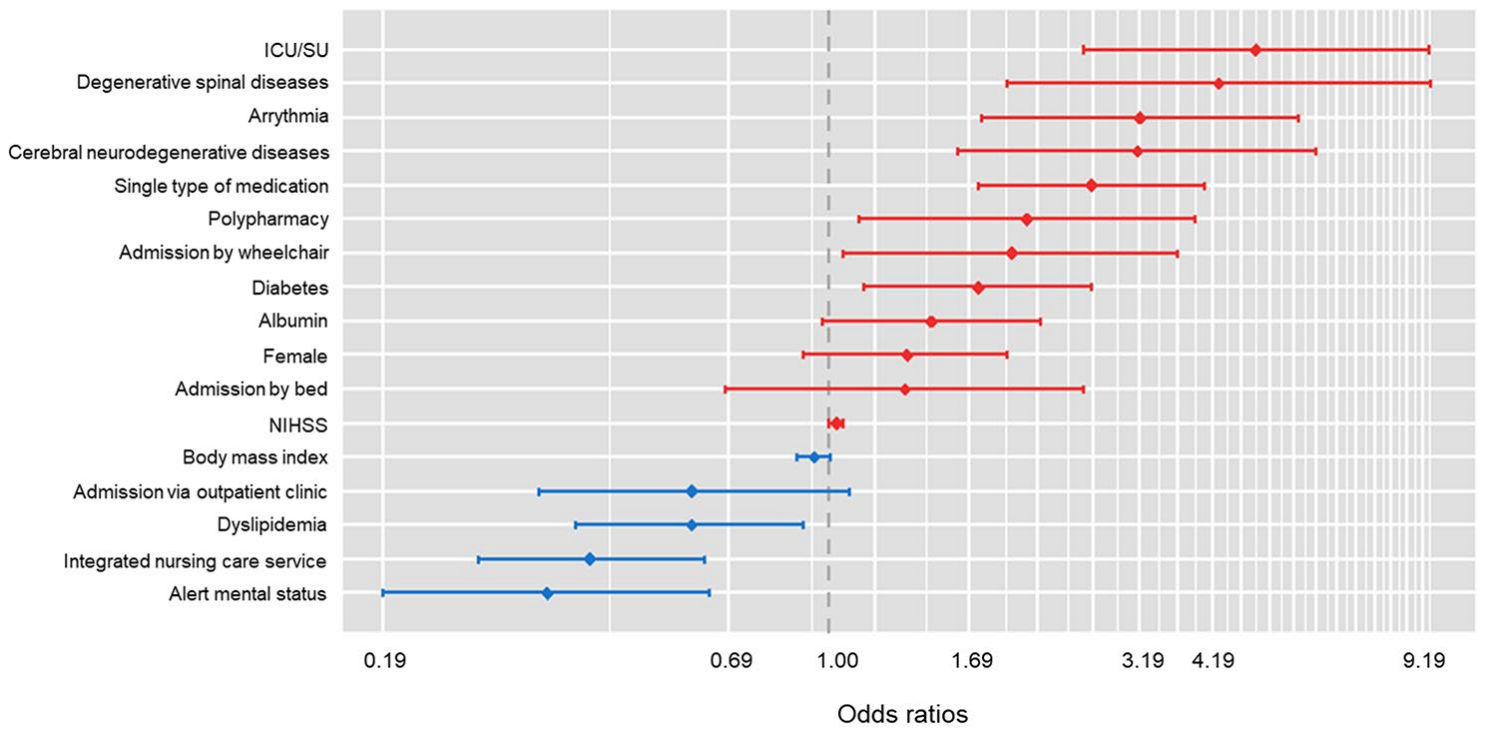
Forest plot of the final stepwise logistic regression model for predicting in-hospital falls

Table 3 presents the final binary logistic regression model after stepwise backward elimination. Figure 3 shows the distribution of adjusted odds ratios (aOR) and 95% confidence intervals (CI) for each variable. The type of ward was significantly associated with a lower risk of falls in the INCS, whereas the ICU/SU was associated with a higher risk of falls. In addition, admission with wheelchair ambulation, diabetes, arrhythmia, degenerative spinal diseases, cerebral neurodegenerative diseases, and medications were significantly associated with a higher risk of falls. In comparison, dyslipidemia and alert mental status were significantly associated with a lower risk of falls.

**Table 3:** Final logistic regression model after stepwise backward elimination

Variables	Odds ratio (95% Confidence interval)	*p*-value
Body mass index	0.95 (0.89–1.01)	0.092
Female	1.34 (0.91–1.95)	0.136
Albumin	1.47 (0.98–2.21)	0.059
Ward type		
General	Reference	
Integrated nursing care service	0.41 (0.27–0.63)	< 0.001
Intensive care unit/stroke unit	4.93 (2.59–9.41)	< 0.001
Admission route		
Emergency department	Reference	
Outpatient clinic	0.60 (0.34–1.08)	0.091
Admission state		
On foot	Reference	
Wheelchair	1.98 (1.06–0.36)	0.032
Bed	1.33 (0.68–2.60)	0.405
Hypertension		
Diabetes	1.75 (1.14–2.67)	0.010
Dyslipidemia	0.60 (0.39–0.91)	0.015
Arrhythmia	3.20 (1.77–5.78)	< 0.001
Degenerative spinal diseases	4.30 (1.95–9.49)	< 0.001
Cerebral neurodegenerative diseases	3.17 (1.62–6.20)	< 0.001
Medication		
None	Reference	
Single	2.67 (1.75–4.07)	< 0.001
Poly	2.10 (1.12–3.94)	0.020
Alert mental status	0.35 (0.19–0.64)	< 0.001

### ML prediction

Variables with zero variance, such as osteoporosis, cardiovascular disease, and degenerative spinal diseases, were excluded from the analysis. No evidence of multicollinearity was noted among the continuous variables with correlation coefficients of ≥ 0.7. The ratios of falls to non-falls in the training and validation datasets were 104:626 and 52:308, respectively. After applying the synthetic minority oversampling technique to the training dataset, the revised ratio of falls to non-falls became 624:626. Table S4 presents the confusion matrix for all prediction models.

Among the six ML algorithms, XGB had the highest AUROC of 0.85 (0.78–0.92), and XGB and NB had the highest F1 score of 0.44. The KNN-based prediction model had the highest sensitivity of 0.71 (0.58–0.82), whereas XGB had the highest sensitivity at 0.65 (0.46–0.81). RF showed the highest positive predictive value of 0.85 (0.58–0.96), and KNN showed the highest negative predictive value of 0.94 (0.90–0.96). All ML algorithms showed similar or slightly improved AUROC values compared with MFS (Table 4).

**Table 4:** Machine learning prediction performance

	RLR	SVM	NB	KNN	RF	XGB
AUROC	0.77 (0.69–0.85)	0.80 (0.73–0.87)	0.76 (0.68–0.84)	0.77 (0.69–0.85)	0.83 (0.76–0.90)	0.85 (0.78–0.92)
F1 score	0.42	0.41	0.44	0.41	0.34	0.44
Sensitivity	0.69 (0.56–0.80)	0.39 (0.26–0.52)	0.50 (0.37–0.63)	0.71 (0.58–0.82)	0.21 (0.12–0.34)	0.33 (0.22–0.46)
Specificity	0.73 (0.68–0.78)	0.92 (0.88–0.94)	0.87 (0.82–0.90)	0.71 (0.65–0.76)	0.99 (0.98–1.00)	0.97 (0.95–0.98)
PPV	0.31 (0.23–0.39)	0.44 (0.30–0.58)	0.39 (0.28–0.51)	0.29 (0.22–0.38)	0.85 (0.58–0.96)	0.65 (0.46–0.81)
NPV	0.93 (0.90–0.96)	0.90 (0.86–0.93)	0.91 (0.87–0.94)	0.94 (0.90–0.96)	0.88 (0.84–0.91)	0.90 (0.86–0.92)

AUROC, area under the receiver operating curve; KNN, *k*-nearest neighbors; NB, naïve Bayes; NPV, negative predictive value; PPV, positive predictive value; RF, random forest; RLR, regularized logistic regression; SVM, support vector machine; XGB, extreme-gradient boosting

**Fig. 4:**
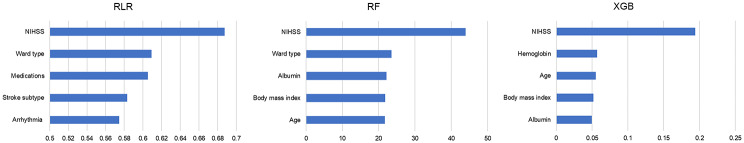
Feature importance analysis revealing the top five most important variables in regularized logistic regression (RLR), random forest (RF), and extreme gradient boosting (XGB) algorithms. Stroke severity was the most important for predicting in-hospital falls

The NIHSS was the most important feature in predicting falls in all models, including RLR, RF, and XGB. Other variables such as age, BMI, albumin, and hemoglobin were also important predictors. Ward type was a significant variable for predicting falls. In addition, medications and arrhythmia were identified as the top five variables in the RLR model (Fig. 4).

## Discussion

This study proposed ML-based models for predicting in-hospital falls in acute stroke using EHRs. The models demonstrated comparable performance to the MFS in predicting falls. Previous studies using ML to predict falls in hospitalized patients have reported valid results [31–33]. Wang et al. [34] reported a robust fall prediction with multi-view ensemble learning with missing values, and their model showed an AUROC of 0.81, which was similar to ours. Nakatani et al. [29] presented a natural language process-based inpatient fall prediction model using EHRs and reported an AUROC of 0.84, which was similar to ours. Our results show that disease-specific variables are essential predictors of falls in this patient group and can improve the accuracy of fall prediction. Furthermore, our findings suggest that ML algorithms can be tailored to specific healthcare settings and disease populations to develop more accurate prediction models. Such prediction models may be critical in reducing fall-related injuries and, ultimately, improving patient outcomes.

Moreover, developing and applying a fall prediction model using ML algorithms has clinical significance in improving the efficiency of medical staff. Nursing staff feel much stress and limitations when assessing and intervening for fall risk with assessment tools [35]. Furthermore, identifying fall risk factors based on the characteristics of each patient requires time and can become an excessive burden [36]. In actual clinical practice, it is difficult for nursing staff to search and find individual risk factors for falls for each patient and provide nursing care accordingly. To overcome these limitations, the use of ML algorithms to predict falls provides an easy and fast way to obtain accurate results. Therefore, this approach has significant clinical significance, enabling nursing staff to predict falls quickly and accurately and intervene accordingly, reducing fall occurrences.

One notable finding among the critical risk factors for falls in patients with acute stroke was the ward type, which was particularly important in INCS. Previous studies in South Korea have yielded inconsistent results regarding the relationship between fall rates and INCS, with some showing higher rates and others showing no significant difference [37, 38]. The present study proved that INCS significantly reduced the risk of falls in patients hospitalized with acute stroke. Thus, the characteristics of patients with acute stroke, most of whom show varying degrees of neurological impairment, may have contributed to these results. In cerebrovascular specialty hospitals, INCS might have focused on fall prevention activities on such disease characteristics. However, more studies are needed to explore this relationship further.

BMI can reflect nutritional status [39], and our results that BMI was one of the critical variables to predict in-hospital falls in patients with acute stroke can indicate that falls may occur frequently in patients with low body weight or weakened physical motor function [40]. Among our results, albumin and hemoglobin levels were found to be important variables for fall risk. Previous studies have reported low albumin levels and anemia as risk factors for falls in patients hospitalized in the acute phase, and these could be equally applied to patients with acute stroke. Finally, socioeconomic status, a well-known risk factor, was found to be unrelated to the in-hospital falls in this study [41, 42]. These results were attributed to the reason that this study was conducted in a single region and incorporated only patients with acute stroke. Therefore, we consider that disease characteristics may make a greater contribution to the risk of falls than socioeconomic characteristics.

Medication use, a well-known fall predictor, was another critical variable in our analysis. Previous studies have shown that medication use, including analgesics, sedatives, vasodilators, and muscle relaxants, is a significant risk factor for falls [43, 44]. Further, polypharmacy increases fall risk [45]. This is particularly relevant for patients with acute stroke because they often have comorbid conditions and receive multiple medications, including central nervous system medications, sedatives, and narcotics, all associated with increased fall risk [46].

In the present study, ensemble models – RF and XGB – showed slightly higher AUROC values but generally lower sensitivity. Conversely, more classical ML algorithms such as the RLR and KNN showed decent AUROC values, along with balanced sensitivity and specificity. This can be attributed to the regularization and relatively simple classification methods overcoming overfitting better than the tree-based ensemble models in this dataset [47]. However, these results cannot be generalized, and more studies based on various databases are needed. Further, this model is intended for screening to prevent falls and is very cost-effective. However, the cost can be much greater once a fall event occurs. Therefore, even if the sensitivity is relatively low, their high specificity and negative predictive value can provide clues for nursing staff to select and focus on patients who need to focus more on fall-prevention activities during their hospitalization [48].

This study is the first to develop an ML-based fall prediction model for patients with acute stroke. We were able to present validated results of ML prediction by comparing them with the MFS, which is the most widely utilized existing fall prediction tool. Furthermore, using multiple ML algorithms for prediction made it possible to directly compare each model’s performance.

This study has several limitations. First, this was a single-center study, which may have limited generalizability. More studies using big data from multiple institutions are needed to verify the results and improve generalizability. Second, this retrospective study used EHRs, which might result in ambiguity in defining some variables. Third, the dataset only observed falls during hospitalization for acute stroke and did not provide long-term follow-up outcomes. Fourth, the timing drug information collection was different between groups. That is, in the fall group, when an event occurred, the medication list was identified with the index date, but in the non-fall group, it was identified with the admission date as the index date. This may have been a source of bias. Finally, despite various statistical adjustments, the outcome variable, in-hospital falls, has a highly imbalanced ground truth, making it difficult to establish causality.

## Conclusions

In this study, the ML algorithms used for predicting in-hospital falls among patients with acute stroke showed valid results. Their prediction performance was not equivalent to that of the MFS and they can be readily applied and overcoming the disadvantages of the MFS at the same time. Furthermore, the ML models integrate initial clinical information in a meaningful direction to enable the construction of prediction models that can be used at the beginning of hospitalization. Therefore, the use of ML models for fall prediction is of great clinical significance in allowing medical staff to perform more accurate and efficient fall screening. Ultimately, this study provided cornerstone data for the practical use of the fall screening model of patients with acute stroke in real clinical settings base.

### Electronic supplementary material

Below is the link to the electronic supplementary material.

Supplementary Material 1

Supplementary Material 2

## Data Availability

The dataset and entire code supporting the conclusions of this article are included within this article and its additional files.

## List of abbreviations

aOR: adjusted odds ratio
AUROC: area under the receiver operating characteristic curve
BMI: body mass index
CI: confidence intervals
EHR: electronic health records
ICU: intensive care unit
INCS: integrated nursing care service
KNN: *k*-nearest neighbors
MFS: Morse fall scale
ML: machine learning
NB: naïve Bayes
RF: random forest
RLR: regularized logistic regression
SU: stroke unit
SVM: support vector machine
XGB: extreme-gradient boosting
