# Supplementary material for: In-hospital fall prediction using machine learning algorithms and the Morse fall scale in patients with acute stroke: a nested case-control study
Source: BMC Med Inform Decis Mak. 2023 Nov 1;23:246. doi: 10.1186/s12911-023-02330-0 (PMC10619231; doi:10.1186/s12911-023-02330-0)
Supplement: Supplementary file 2 — Supplementary Material 2 [file 12911_2023_2330_MOESM2_ESM.docx]

**Supplementary materials**

**Table S1.** Definition of comorbidities.

**Table S2.** List of variables for machine learning prediction.

**Table S3.** Tuned hyperparameters for each machine learning algorithm.

**Table S4.** Confusion matrix for each prediction.

**R codes for this study** (The R Project for Statistical Computing; https://www.R-project.org/).

**Table S1.** Definition of comorbidities in this study.

| **Comorbidities** | **ICD-10 codes** |
| --- | --- |
| Hypertension | I10-14 |
| Diabetes | E10-14 |
| Dyslipidemia | E68 |
| Arrhythmia | I46-49 |
| Coronary artery disease | I20-24 |
| Osteoporosis | M80-82 |
| Degenerative spinal diseases | M43, M44-44 |
| Cerebral neurodegenerative diseases |  |
| Parkinsonism | G20-22 |
| Dementia | F00, F01, F02, G30, G31 |
| Multiple system atrophy | G23 |
| Multiple sclerosis | G34 |

**Table S2.** List of variables for machine learning prediction.

| **Fall risk category** | | **Research variables** | **Source** |
| --- | --- | --- | --- |
| Fall characteristics | | Place, Injury, Time, Day | Fall occurrence report |
| Fall risk assessment tool | | MFS | Fall assessment record |
| Fall risk  factor | Patient  characteristics | Age, Body mass index, Sex,  Insurance types,  Residential areas | First-time nursing record |
|  | Pathophysiologic and disease-related  factors | Hemoglobin, Albumin,  Stroke types  NIHSS | Laboratory test result data  Doctor’s note  NIHSS record |
|  | Therapeutics | Comorbidities | Diagnostic codes |
|  |  | Medications | Drug prescription data  Nursing and doctor’s records |
|  | Mental and cognitive  factors | Mental state | Nursing and doctor’s records |
|  | Situational factors | Ward types  Admission route and state | First-time nursing record |

MFS, Morse Fall scale; NIHSS, National Institutes of Health Stroke scale.

**Table S3.** Tuned hyperparameters for each machine learning algorithm.

| **Algorithms** | **Tuned Hyperparameters** |
| --- | --- |
| RLR | cost = 4; loss = L1; epsilon = 0.1 |
| SVM | sigma = 0.01669972; C = 4 |
| NB | fL = 0; usekernel = TRUE; adjust = 1.0 |
| KNN | k = 7 |
| RF | mtry = 2; ntree=1000 |
| XGB | nrounds = 1000; max_depth = 8; eta = 0.01; gamma = 0; colsample_bytree = 0.6; min_child_weight = 1; subsample = 0.8 |

KNN, *k*-nearest neighbors; NB, Naïve bayes; RF, random forest; RLR, regularized logistic regression; SVM, support vector machines; XGB, extreme gradient boosting.

**Table S4.** Confusion matrix for each prediction.

| **Models** | **Prediction** | **Reference** | |
| --- | --- | --- | --- |
|  |  | Non-fall | Fall |
| RLR | Non-fall | 226 | 16 |
|  | Fall | 82 | 36 |
| SVM | Non-fall | 282 | 32 |
|  | Fall | 26 | 20 |
| NB | Non-fall | 267 | 26 |
|  | Fall | 41 | 26 |
| KNN | Non-fall | 218 | 15 |
|  | Fall | 90 | 37 |
| RF | Non-fall | 306 | 41 |
|  | Fall | 2 | 11 |
| XGB | Non-fall | 299 | 35 |
|  | Fall | 9 | 17 |

KNN, *k*-nearest neighbors; NB, Naïve bayes; RF, random forest; RLR, regularized logistic regression; SVM, support vector machines; XGB, extreme gradient boosting.

R codes for this study (The R Project for Statistical Computing; https://www.R-project.org/).

library(caret)
library(data.table)
library(dplyr)
library(LiblineaR)
library(e1071)
library(kernlab)
library(randomForest)
library(tidyverse)
library(xgboost)
library(smotefamily)
library(klaR)
library(GGally)
library(MLeval)
library(moonBook)
library(pROC)
library(doParallel)

fall<- read_csv("new.csv")
SF<-na.omit(fall)

SF$sex <- as.factor(SF$sex)
SF$ward <- as.factor(SF$ward)
SF$ins <- as.factor(SF$ins)
SF$reg <- as.factor(SF$reg)
SF$type <- as.factor(SF$type)
SF$route <- as.factor(SF$route)
SF$admway <- as.factor(SF$admway)
SF$htn <- as.factor(SF$htn)
SF$dia <- as.factor(SF$dia)
SF$dl <- as.factor(SF$dl)
SF$arr <- as.factor(SF$arr)
SF$cad <- as.factor(SF$cad)
SF$osteo <- as.factor(SF$osteo)
SF$degspine <- as.factor(SF$degspine)
SF$nd <- as.factor(SF$nd)
SF$ms <- as.factor(SF$ms)
SF$med <- as.factor(SF$med)
SF$class <- as.factor(SF$class)

mytable(class~., data=SF)

predata<-SF
nearZeroVar(predata)

predata$osteo=NULL
predata$cad=NULL
predata$degspine=NULL
predata$score=NULL
predata$mrs=NULL
predata$mbi=NULL

findCorrelation(cor(predata[,2:6]), cutoff = .7)

ggpairs(predata[,2:6], lower=list(continuous="smooth"))


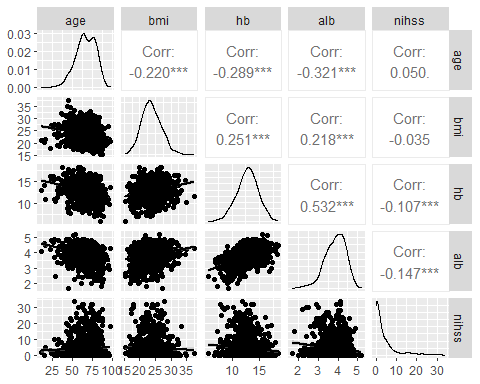


st_model<-preProcess(predata[,2:6], method=c("center","scale"))
data<-predict(st_model, predata)
data=as.data.frame(data)

ohe_feats=c('sex','ward','ins','reg','type','route','admway','htn','dia','dl','arr','nd','ms','med')

dummies=dummyVars(~sex+ward+ins+reg+type+route+admway+htn+dia+dl+arr+nd+ms+med, data = data)

df_ohe <- as.data.frame(predict(dummies, newdata = data))
df_combined <- cbind(data[,-c(which(colnames(data) %in% ohe_feats))],df_ohe)
dat = as.data.table(df_combined)

table(dat$class)

seed<-1111
set.seed(seed)
ind<-sample(2,nrow(dat),replace = T,prob = c(0.67,0.33))
traindata<-dat[ind==1,]
testdata<-dat[ind==2,]

table(traindata$class)

table(testdata$class)

set.seed(seed)
traindata.smote <- SMOTE(traindata[,-1], traindata$class ,K = 5, dup_size=0)
traindata.smote <- traindata.smote$data
traindata.smote$class <- as.factor(traindata.smote$class)
table(traindata.smote$class)

traindata.smote$Newclass<-relevel(traindata.smote$class, ref = "N")
levels(traindata.smote$Newclass)

traindata.smote$class=NULL

detectCores()

cl <- makePSOCKcluster(12)
registerDoParallel(cl)

ctrl <- trainControl(method = "repeatedcv", number = 10, repeats = 10, savePredictions = TRUE, classProbs = TRUE, summaryFunction = twoClassSummary, allowParallel=TRUE)

metric <- "ROC"

set.seed(seed)
lr_fit<-train(class~., data = traindata, method = "regLogistic", trControl=ctrl, metric=metric, tuneLength=5, verbose=FALSE)
lr_fit

xlr <- evalm(lr_fit)

xlr$stdres

test_pred_lr <- predict(lr_fit, newdata = testdata)
confusionMatrix(test_pred_lr, testdata$class)

test_prob_lr <- predict(lr_fit, newdata = testdata, type="prob")

mlr = data.frame(test_prob_lr, testdata$class)
ylr<-evalm(mlr)

ylr$stdres

imp<-varImp(lr_fit, scale = FALSE)
imp

plot(imp)

set.seed(seed)
svm_fit<-train(class~., data = traindata, method = "svmRadial", trControl=ctrl, metric=metric, tuneLength=5, verbose=FALSE)

xsvm <- evalm(svm_fit)

xsvm$stdres

test_pred_svm <- predict(svm_fit, newdata = testdata)
confusionMatrix(test_pred_svm, testdata$class)

test_prob_svm <- predict(svm_fit, newdata = testdata, type="prob")

msvm = data.frame(test_prob_svm, testdata$class)
ysvm<-evalm(msvm)

ysvm$stdres

set.seed(seed)
knn_fit <- train(class~., data = traindata, method = "knn", trControl=ctrl, metric=metric, tuneLength=5)
knn_fit

x_knn <- evalm(knn_fit)

x_knn$stdres

test_pred_knn <- predict(knn_fit, newdata = testdata)
confusionMatrix(test_pred_knn, testdata$class)

test_prob_knn <- predict(knn_fit, newdata = testdata, type="prob")
mknn = data.frame(test_prob_knn, testdata$class)
yknn<-evalm(mknn)

yknn$stdres

set.seed(seed)
nb_fit <- train(class~., data = traindata, method = "nb", trControl=ctrl, metric=metric, tuneLength=5, verbose=FALSE)
nb_fit

x_nb <- evalm(nb_fit)

x_nb$stdres

test_pred_nb <- predict(nb_fit, newdata = testdata)
confusionMatrix(test_pred_nb, testdata$class)

test_prob_nb <- predict(nb_fit, newdata = testdata, type="prob")
mnb = data.frame(test_prob_nb, testdata$class)
ynb<-evalm(mnb)

ynb$stdres

set.seed(seed)
rf_fit <- train(class~., data = traindata, method = "rf", trControl=ctrl, metric=metric, tuneLength=5, ntree=1000, verbose=FALSE)
rf_fit

x_rf <- evalm(rf_fit)

x_rf$stdres

test_pred_rf <- predict(rf_fit, newdata = testdata)
confusionMatrix(test_pred_rf, testdata$class)

test_prob_rf <- predict(rf_fit, newdata = testdata, type="prob")
mrf = data.frame(test_prob_rf, testdata$class)
yrf<-evalm(mrf)

yrf$stdres

imp<-varImp(rf_fit, scale = FALSE)
imp

plot(imp)

xgbgrid <- expand.grid(nrounds= c(1000, 1500), max_depth=c(6, 8), eta=c(0.1, 0.01), gamma=0, colsample_bytree=0.6, min_child_weight=1, subsample=0.8)

set.seed(seed)
xgb_fit <- train(class~., data = traindata, method = "xgbTree", trControl=ctrl, metric=metric, tuneGrid=xgbgrid, ntree=1000, verbose=FALSE)

xgb_fit

x_xgb <- evalm(xgb_fit)

x_xgb$stdres

test_pred_xgb <- predict(xgb_fit, newdata = testdata)
confusionMatrix(test_pred_xgb, testdata$class)

test_prob_xgb <- predict(xgb_fit, newdata = testdata, type="prob")
mxgb= data.frame(test_prob_xgb, testdata$class)
yxgb<-evalm(mxgb)

yxgb$stdres

imp<-varImp(xgb_fit, scale = FALSE)
imp

plot(imp)

set.seed(seed)
lr_fit<-train(Newclass~., data = traindata.smote, method = "regLogistic", trControl=ctrl, metric=metric, tuneLength=5, verbose=FALSE)
lr_fit

xlr <- evalm(lr_fit)

xlr$stdres

test_pred_lr <- predict(lr_fit, newdata = testdata)
confusionMatrix(test_pred_lr, testdata$class)

test_prob_lr <- predict(lr_fit, newdata = testdata, type="prob")

mlr = data.frame(test_prob_lr, testdata$class)
ylr<-evalm(mlr)

ylr$stdres

imp<-varImp(lr_fit, scale = FALSE)
imp

plot(imp)

set.seed(seed)
svm_fit<-train(Newclass~., data = traindata.smote, method = "svmRadial", trControl=ctrl, metric=metric, tuneLength=5, verbose=FALSE)
svm_fit

xsvm <- evalm(svm_fit)

xsvm$stdres

test_pred_svm <- predict(svm_fit, newdata = testdata)
confusionMatrix(test_pred_svm, testdata$class)

test_prob_svm <- predict(svm_fit, newdata = testdata, type="prob")

msvm = data.frame(test_prob_svm, testdata$class)
ysvm<-evalm(msvm)

ysvm$stdres

set.seed(seed)
knn_fit <- train(Newclass~., data = traindata.smote, method = "knn", trControl=ctrl, metric=metric, tuneLength=5)
knn_fit

x_knn <- evalm(knn_fit)

x_knn$stdres

test_pred_knn <- predict(knn_fit, newdata = testdata)
confusionMatrix(test_pred_knn, testdata$class)

test_prob_knn <- predict(knn_fit, newdata = testdata, type="prob")
mknn = data.frame(test_prob_knn, testdata$class)
yknn<-evalm(mknn)

yknn$stdres

set.seed(seed)
nb_fit <- train(Newclass~., data = traindata.smote, method = "nb", trControl=ctrl, metric=metric, tuneLength=5, verbose=FALSE)
nb_fit

x_nb <- evalm(nb_fit)

x_nb$stdres

test_pred_nb <- predict(nb_fit, newdata = testdata)
confusionMatrix(test_pred_nb, testdata$class)

test_prob_nb <- predict(nb_fit, newdata = testdata, type="prob")
mnb = data.frame(test_prob_nb, testdata$class)
ynb<-evalm(mnb)

ynb$stdres

set.seed(seed)
rf_fit <- train(Newclass~., data = traindata.smote, method = "rf", trControl=ctrl, metric=metric, tuneLength=5, ntree=1000, verbose=FALSE)
rf_fit

x_rf <- evalm(rf_fit)

x_rf$stdres

test_pred_rf <- predict(rf_fit, newdata = testdata)
confusionMatrix(test_pred_rf, testdata$class)

test_prob_rf <- predict(rf_fit, newdata = testdata, type="prob")
mrf = data.frame(test_prob_rf, testdata$class)
yrf<-evalm(mrf)

yrf$stdres

imp<-varImp(rf_fit, scale = FALSE)
imp

plot(imp)

xgbgrid <- expand.grid(nrounds= c(1000, 1500), max_depth=c(6, 8), eta=c(0.1, 0.01), gamma=0, colsample_bytree=0.6, min_child_weight=1, subsample=0.8)

set.seed(seed)
xgb_fit <- train(Newclass~., data = traindata.smote, method = "xgbTree", trControl=ctrl, metric=metric, tuneGrid=xgbgrid, ntree=1000, verbose=FALSE)

xgb_fit

x_xgb <- evalm(xgb_fit)

x_xgb$stdres

test_pred_xgb <- predict(xgb_fit, newdata = testdata)
confusionMatrix(test_pred_xgb, testdata$class)

test_prob_xgb <- predict(xgb_fit, newdata = testdata, type="prob")
mxgb= data.frame(test_prob_xgb, testdata$class)
yxgb<-evalm(mxgb)

yxgb$stdres

imp<-varImp(xgb_fit, scale = FALSE)
imp

plot(imp)
